# Animal-Assisted Intervention for trauma: a systematic literature review

**DOI:** 10.3389/fpsyg.2015.01121

**Published:** 2015-08-07

**Authors:** Marguerite E. O'Haire, Noémie A. Guérin, Alison C. Kirkham

**Affiliations:** Center for the Human-Animal Bond, Center for Animal Welfare Science, Department of Comparative Pathobiology, College of Veterinary Medicine, Purdue UniversityWest Lafayette, IN, USA

**Keywords:** Animal-assisted Intervention, animal-assisted therapy, child abuse, human-animal interaction, posttraumatic stress disorder, trauma, veteran

## Abstract

Animals have a long history of inclusion in psychiatric treatment. There has been a recent growth in the empirical study of this practice, known as Animal-Assisted Intervention (AAI). We conducted a systematic review of the empirical literature on AAI for trauma, including posttraumatic stress disorder (PTSD). Ten studies qualified for inclusion, including six peer-reviewed journal articles and four unpublished theses. Participants were predominantly survivors of child abuse, in addition to military veterans. The presentation of AAI was highly variable across the studies. The most common animal species were dogs and horses. The most prevalent outcomes were reduced depression, PTSD symptoms, and anxiety. There was a low level of methodological rigor in most studies, indicating the preliminary nature of this area of investigation. We conclude that AAI may provide promise as a complementary treatment option for trauma, but that further research is essential to establish feasibility, efficacy, and manualizable protocols.

## Introduction

The inclusion of animals in psychological treatment is not new, nor is it uncommon. The first reported occurrence is estimated to be the late eighteenth century, when animals were incorporated into mental health institutions to increase socialization among patients (Serpell, 2006). Today, a number of programs in the United States report involving animals in their services in some capacity. One of the most commonly targeted populations for these services is individuals who have experienced trauma, including those with posttraumatic stress disorder (PTSD; Tedeschi et al., 2010). Yet despite the popularity of positive media surrounding these programs, it is unclear whether empirical data supports their practice. The purpose of this review is to systematically collect and critically assess current research on Animal-Assisted Intervention (AAI) for trauma, including PTSD.

AAI is broadly defined as any intervention that includes an animal as part of the process (Kruger and Serpell, 2010). It encompasses targeted therapeutic interventions with animals (Animal-Assisted Therapy), less structured enrichment activities with animals (Animal-Assisted Activities), and the provision of trained animals to assist with daily life activities (Service or Assistance Animals). The use of AAI has been related to promising outcomes in a number of populations, including increased social interaction among children with autism spectrum disorder (O'Haire, 2013), increased social behaviors and reduced agitation and aggression among persons with dementia (Filan and Llewellyn-Jones, 2006; Bernabei et al., 2013), reduction in symptoms among patients with depression (Souter and Miller, 2007), and increased emotional well-being such as reduced anxiety and fear (Nimer and Lundahl, 2007). It is purported to provide value for trauma in similar ways.

PTSD is an anxiety disorder that is characterized by symptoms related to intrusion, avoidance, negative alterations in cognition and mood, and alterations in arousal and reactivity (American Psychiatric Association, 2000). It is estimated to affect approximately 7.8% of the US population (Kessler et al., 1995) and can lead to substantial work and social impairments (e.g., Hidalgo and Davidson, 2000). It is a difficult disorder to treat, with dropout and non-response rates up to 50% in studies of empirically-supported treatments (Schottenbauer et al., 2008). One of the most well-established treatments in research, exposure therapy, is not commonly undertaken by therapists due to its perceived level of difficulty and discomfort to patients (e.g., Becker et al., 2004). Discovering and evaluating alternative and complementary therapies has been deemed imperative (Cukor et al., 2009; Bomyea and Lang, 2012).

Anecdotal evidence suggests that animals may provide unique elements to address several PTSD symptoms. With respect to intrusion, the presence of an animal is purported to act as a comforting reminder that danger is no longer present (Yount et al., 2013) and to act as a secure base for mindful experiences in the present (Parish-Plass, 2008). Individuals with PTSD often experience emotional numbing, yet the presence of an animal has been reported to elicit positive emotions and warmth (e.g., Marr et al., 2000; O'Haire et al., 2013). Animals have also been demonstrated as social facilitators that can connect people (e.g., McNicholas and Collis, 2000; Wood et al., 2005) and reduce loneliness (e.g., Banks and Banks, 2002), which may assist individuals with PTSD to break out of isolation and connect to the humans around them. One of the most challenging aspects of PTSD tends to be hyperarousal. The presence of an animal has been linked to secretion of oxytocin (Beetz et al., 2012b) and reductions in anxious arousal (e.g., Barker et al., 2003), which may be a particularly salient feature for individuals who have experienced trauma. Yet despite the theoretical promise of AAI and its popularization through anecdotal media, there has been no comprehensive review of its empirical research base for trauma.

The purpose of this review is to surpass anecdotal accounts by presenting a comprehensive overview of empirical research on AAI for trauma. The goal is to systematically identify, summarize, and evaluate any existing empirical studies of AAI for trauma in order to document currently researched AAI practices and their reported findings, as well as to provide directions for further, more rigorous research. The specific aims are to: (a) describe the characteristics of AAI for trauma, (b) evaluate the state of the evidence base, and (c) summarize the reported outcomes of AAI for trauma.

## Methods

### Protocol

The preferred reporting items for systematic reviews and meta-analyses (PRISMA) guidelines were consulted to perform this systematic review (Liberati et al., 2009; Moher et al., 2009). The study procedures were defined a priori in a study protocol that specified the search strategy, inclusion and exclusion criteria, and data extraction items.

### Eligibility criteria

The following inclusion criteria were used to select relevant articles for review: (a) publication in English in a peer-reviewed journal or thesis, (b) collection of original, empirical data on outcomes from AAI, which was defined as any intervention that intentionally incorporated a live animal, and (c) reporting of summative results for participants who have experienced trauma, including PTSD.

### Search procedure

Studies were identified by searching the following electronic databases from their inception date through October 2014: *ERIC* (1966-Present), *Medline* (1950-Present), *ProQuest* (1971-Present), *PsycARTICLES* (1987-Present), *PsycINFO* (1806-Present), and *Scopus* (1960-Present). To increase coverage, two additional databases were included: *HABRI Central* (Human-Animal Bond Research Initiative), a specialized human-animal interaction research database, and *PILOTS* (Published International Literature on Traumatic Stress), a specialized PTSD research database maintained by the National Center for PTSD of the Department of Veteran Affairs. Search terms for all databases included at least one identifier for trauma and at least one identifier for AAI in the article title, abstract, and/or keywords. Identifiers for trauma included PTSD OR trauma^*^, where the asterisk indicates any word variation including the base “trauma.” Identifiers for AAI included a comprehensive list of 38 search terms used in a previous systematic review of AAI for autism spectrum disorder, detailed in Table 1 (O'Haire, 2013). To reduce overestimation of effects due to potential publication bias, theses were included in addition to peer-reviewed journal articles. For all articles meeting the inclusion criteria, reference lists were screened for possible additions.

**Table 1:** **List of terms to identify Animal-Assisted Intervention (AAI) in database search**.

Animal intervention	Canine therapy	Dolphin assisted	Human animal interaction(s)	Therapeutic animal(s)
Animal therapy	**Canine assisted**	Dolphin facilitated	Pet therapy	Therapeutic dog(s)
**Animal assisted**	Canine facilitated	Equine therapy	Pet assisted	Therapeutic horse(s)
Animal facilitated	Companion animal(s)	Equine assisted	Pet facilitated	Therapeutic horseback
Anthrozoology	Dog therapy	**Equine facilitated**	Service animal(s)	Therapeutic pet(s)
Assistance animal(s)	**Dog assisted**	Hippotherapy	**Service dog(s)**	Therapeutic riding
Assistance dog(s)	Dog facilitated	Horseback riding	Service horse(s)	Therapy with animals
Assistance horse(s)	Dolphin therapy	Human animal bond		

*Bold terms indicate words used to identify AAI in the final review sample*.

### Data extraction and evaluation

Information was extracted from each included study to achieve the three aims of this systematic review. To achieve the first aim—describe key characteristics of the AAIs—data items included AAI terminology, animals, setting, interventionist, format, activities, and duration. To achieve the second aim—evaluate study methodology and risk of bias—data items included sample size, participant characteristics (including age, gender, and PTSD diagnosis), study design, comparison condition, and assessment measures (including type, standardized instruments, and raters/informants). To achieve the third aim—summarize study outcomes—data items included the results of each study, which were subsequently organized by the most commonly reported outcomes. Additional data items were extracted for study identification and exploratory purposes, including first author, publication year, country of corresponding author, and journal name.

To compare effect sizes across studies, we calculated Cohen's *d* for all studies which reported means and standard deviations. We used two different formulas based on study design. For within-participant designs, effect size was calculated by dividing the difference of the means by the average standard deviation of both repeated measures (Lakens, 2013).

(1) $$d_{within}=\frac{M_{\text{diff}}}{\frac{SD_{1}+SD_{2}}{2}}$$

For between-participants designs, effect size was calculated using the recommended formula for pre-post-control group designs using the pooled pre-test standard deviation (Morris, 2007).

(2) $$d_{between}=c_{p}\left[\frac{\left(M_{post,T}-M_{pre,T}\right)-\left(M_{post,C}-M_{pre,C}\right)}{SD_{pre}}\right]$$

Where, the pooled standard deviation is defined as:

(3) $$SD_{pre}=\sqrt{\frac{\left(n_{T}-1\right)SD_{pre,T}^{2}+\left(n_{c}-1\right)SD_{pre,C}^{2}}{n_{T}+n_{C}-2}}$$

and

(4) $$c_{p}\text{=1}-\frac{3}{\text{4}\left(n_{T}+n_{c}-2\right)-1}$$

## Results

### Study selection

**Figure 1:**
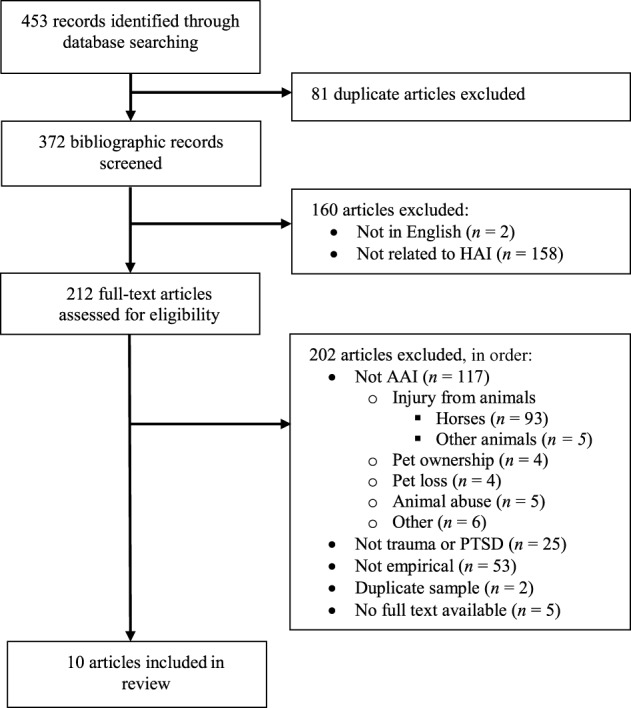
**Flow chart of study selection process**. HAI, human animal interaction; AAI, animal-assisted intervention; PTSD, posttraumatic stress disorder.

The initial literature search resulted in 453 citations. A flow diagram of the study selection process is presented in Figure 1. The final sample included 10 studies (2.2% of the total initial pool) that met the inclusion criteria of empirically evaluating AAI for trauma. Six studies were published in peer-reviewed journals and four were theses. Publication dates ranged from 2004 to 2014, with the majority of studies (9 of 10) being published within the last 4 years, since 2011.

Despite the limitation to English-language articles only, there was an international representation of researchers. Among the peer-reviewed journal articles, countries of corresponding authors included the USA (2 studies), Spain, Germany, Israel, and Australia (1 study each). The articles were primarily published in child or family studies journals (4 studies), in addition to psychology and alternative medicine (1 study each).

The final sample of articles included studies with a range of designs, participant age groups, intervention types, and outcome measures. Due to heterogeneity across studies, the results of this review focus on descriptive and qualitative synthesis rather than meta-analysis.

### Characteristics of AAI for trauma

To achieve the first aim—to describe the characteristics of AAI for trauma—the key features of AAI in the 10 studies were extracted and are summarized in Table 2.

**Table 2:** **Overview of Animal-Assisted Intervention (AAI) characteristics**.

**First author**	**Year**	**AAI terminology**	**Animal**	**Setting**	**Format**	**Interventionist**	**Sessions**		
							**Duration (weeks)**	**Number**	**Length (minutes)**
Woolley^a^	2004	*Animal-assisted therapy*	Horse, Dog, Cat, Rabbit, Farm animals	Farm	Individual and Group	Social workers, Volunteers	9	9	60
Hamama	2011	*Canine-assisted therapy, Dog-assisted therapy*	Dog	School	Group of 9	Therapist, Social workers	12	12	180
McCullough^a^	2011	*Equine-facilitated psychotherapy*	Horse	Riding center	Individual	Therapist, Riding instructor	8	8	90–120
Dietz	2012	*Animal-assisted therapy*	Dog	Treatment center	Group of 6–10	Therapist	7	12	–
Nevins	2013	*Natural horsemanship*	Horse	Riding center	Individual	–	1	4	240
Murrow^a^	2013	*Animal-assisted therapy*	Dog	Treatment center	Individual	Therapist, Dog handler	4	4	15–20
Kemp	2013	*Equine facilitated therapy*	Horse	Riding center	Group	Therapist	9–10	9–10	90
Balluerka	2014	*Animal-assisted therapy*	Horse, Dog, Cat, Farm animals	Farm	Individual and Group of 6–8	Psychologist, Veterinarian	12	34	–
Lass-Hennemann	2014	–	Dog	Laboratory	Individual	Researcher	1	1	20
Newton^a^	2014	*Psychiatric service dog*	Dog	Home	n/a	n/a	>52	n/a	n/a

*Information is reported for the AAI condition only, not any comparison conditions. −, not reported; n/a, not applicable*.

a *unpublished thesis*.

#### Terminology

The terminology used to identify AAI varied depending on the type of animal participating in the study. Seven different terms were used across the 10 studies, with “animal-assisted therapy” occurring the most frequently (*n* = 4). Other terms included “equine-facilitated therapy” (*n* = 1), “natural horsemanship” (*n* = 1) and “psychiatric service dogs” (*n* = 1). One study incorporated two different terms, using both “dog-assisted therapy” and “canine-assisted therapy” in different instances. A final study did not include a specific term to denote AAI other than reporting the “presence of a dog.”

#### Animals and settings

The majority of studies included dogs as the participating species (*n* = 5), while other studies focused on horses (*n* = 3), or a combination of dogs, horses, and other farm animals (*n* = 2). All horse-based interventions took place at a riding facility. The dog-based interventions occurred in a variety of settings, including treatment centers (*n* = 2), a school (*n* = 1), a laboratory (*n* = 1), and the participant's home (*n* = 1).

In the studies that included information about the participating animals' background (*n* = 9), four studies classified the animal as a “therapy dog” or “service dog” that had received prior training (Dietz et al., 2012; Murrow, 2013; Lass-Hennemann et al., 2014; Newton, 2014). Two of these studies specified the therapy dog training organization. One received certification from Therapy Dogs International (Murrow, 2013) and the other was trained at Therapiehundezentrum Saar (Lass-Hennemann et al., 2014). In the remaining studies (*n* = 5), the animals were reported to have had prior socialization with humans, but no specified training.

#### Interventionists and format

All but two of the studies (*n* = 8) included specified interventionists who facilitated the AAI sessions. Over half of these studies included an interventionist who had previous experience or training in AAI (*n* = 5); however, the specifics of their background are not mentioned. At least one therapist, psychologist, or social worker was present for most of the studies (*n* = 6), and was often accompanied by an animal professional, including a dog handler (*n* = 2), riding instructor (*n* = 1), or veterinarian (*n* = 1). In the remaining two studies, one involved a service dog so it did not require an interventionist and the other included the researcher as the study facilitator in a laboratory setting.

The format of the AAIs included individual sessions, group sessions, and a combination of both forms. The individual format used a triangle approach with the participant, the interventionist(s), and the animal (*n* = 3). The group and mixed formats included varying numbers of participants in each group, with only two of four studies reporting group size (range: 6–10).

#### Activities

The activities and role of the interventionist were inconsistently described with varying levels of detail. No studies reported the use of a published, manualized protocol. Most reported the procedures undertaken within the text of the publication; however, none provide enough detail to allow for replication. Three studies reported having a predetermined theme for each session. Among the activities described, two main variation factors were identified: the animal species, and whether or not the intervention animal was used as a metaphor for the child's relationship with his or her usual social partners.

Variation in activity based on animal species may have been due to the nature of indoor vs. outdoor animals. Dogs were the only species included in traditional, clinic-based therapy sessions, whereas horses and farm animals tended to be part of more active engagement outdoors. Three of five studies with dogs integrated them into classical therapy sessions, which included both dog-focused activities such as training as well as talking to the dog about personal traumatic experiences (Hamama et al., 2011; Dietz et al., 2012; Murrow, 2013). Only one study evaluated the effect of different components of the AAI, comparing the effect of the mere presence of the dog vs. the integration of the dog through stories told from the animal's perspective (Dietz et al., 2012). The effects of the dog were generally enhanced by telling a therapeutic story about the dog, which may be attributed to giving the dog a role and integrated purpose in the therapy session, rather than being a mere entity in the room.

In the three studies involving horses as the sole animal, only one included horse riding (McCullough, 2011), while the other two used ground-based activities, reported as “basic horsemanship” and “natural horsemanship.” Examples of ground-based activities included grooming, observing horse behavior, and using body language to direct the horse around the pen. One study also provided education about horse biology and behavior without the horses present.

The two interventions on farm settings incorporated a variety of farm animals in addition to horses and dogs, such as cats, sheep, pigs, chickens, opossums, and llamas. The activities in these programs were not standardized and varied greatly. In one study, participants were given free time to interact with the animals and engage in various activities, such as dog training or gardening (Woolley, 2004). In the other study, participants chose one animal at the beginning of the program to interact preferentially with throughout the program (Balluerka et al., 2014). Neither study reported which animals were most often selected, nor the frequency or duration of interacting with different species.

Among the 10 studies, two did not report the activities undertaken due to the nature of the intervention. The first was based in a laboratory setting, where the AAI consisted of the simple presence or absence of a dog. The second involved the provision of service dogs in the participants' homes. Across the eight remaining studies, seven used human-animal interaction as a metaphor for the participant's relationship with his or her usual social partners. This type of metaphor was incorporated into all AAIs with horses, both AAIs on farm settings, and two AAIs with dogs (Hamama et al., 2011; Murrow, 2013).

#### Duration

Eight studies applied programs with ongoing sessions. These AAIs lasted from 1 to 12 weeks. Most reported an exact duration; however, if only a range was reported, we used the midpoint of the range in descriptive calculations. The average duration of AAI was 7.8 weeks (range: 1–12, *SD* = 3.5) with 11.6 sessions (range: 4–34, *SD* = 9.0), each lasting 115.4 min (range: 17.5–2 40, *SD* = 74.3). The duration of AAI in the remaining two studies included a laboratory-based study with a single, 20-min session, and interviews about service dogs, who had lived with participants for at least 1 year.

### Methodological evaluation

To achieve the second aim, to evaluate study methodology and risk of bias, key characteristics of the methods were extracted and summarized with respect to each study's sample size and characteristics, study design (Table 3), and assessment type (Table 4).

**Table 3:** **Summary of participants, study design, and outcomes**.

**First author**	**Year**	**Participants**				**Study design**	**Comparison condition(s)**	**Outcomes at post-AAI**
		***N***	**Age (years)**	**Gender (% male)**	**Type of trauma**			
Woolley^a^	2004	21	11–17	52	Abuse	Non-randomized control	Residential care without farm visits	↓ Anxiety (pre-session^*^, pre-AAI−, post-comparison−) ↓ Depression (pre-session^*^^b^, pre-AAI−, post-comparison−) − Global functioning
Hamama	2011	9	14–16	0	Abuse	Pre-post	None	↓ PTSD symptoms (pre-AAI^*^) ↓ Depression (pre-AAI^^†^^) − Coping, subjective well-being
McCullough^a^	2011	11	10–18	55	Abuse	Pre-post	None	↓ PTSD symptoms (pre-AAI^*^) ↑ Human-animal bond (pre-AAI^*^)
Dietz	2012	153	7–17	7	Abuse	Non-randomized control	Group therapy without animal	↓ PTSD symptoms (pre-AAI^***^, post-comparison−) ↓ Depression (pre-AAI^***^, post-comparison^***^) ↓ Anxiety, dissociation, anger (pre-AAI^***^, post-comparison^**^) ↓ Sexual concerns (pre-AAI^**^, post-comparison^*^)
Nevins	2013	1	52	100	War	Pre-post	None	↓ PTSD symptoms, depression, dissatisfaction ↑ Happiness, resilience, satisfaction, sleep, social support
Murrow^a^	2013	9	4–12	33	Abuse	Pre-post	None	↑ Approach behaviors toward the dog − Social-emotional competencies
Balluerka	2014	46	12–17	70	Abuse	Non-randomized control	Residential care without farm visits	↑ Attachment security (pre-AAI^*^, post-comparison−) − Trauma, family concern, parental interference, self-sufficiency
Kemp	2013	30	8–17	20	Abuse	AB	Individual counseling without animal	↓ Depression, maladaptive behavior (pre-AAI ^***^, post-comparison^**^) for children ↓ PTSD symptoms, anxiety, depression, dissociation, sexual concerns (pre-AAI ^***^, post-comparison^**^) for adolescents
Lass-Hennemann	2014	80	–	0	Video	Randomized control	Stuffed dog, person, or alone	↓ Anxiety, negative affect (alone^**^, stuffed dog^*^, person−) − Blood pressure, cortisol level, heart rate, positive affect
Newton^a^	2014	6	–	83	War	–	None	↓ Depression, fear of public spaces, medication use, nightmares ↑ Outreach

AAI, Animal-Assisted Intervention; PTSD, Posttraumatic Stress Disorder; −, not reported; Pre-post, simple pre-test and post-test only; AB, waitlist + treatment; ↑ greater, ↓ less, − minimal/no change at post-AAI compared to pre-session (comparison of before to after individual AAI sessions), pre-AAI (comparison of before to after the AAI program in its entirety), and/or post-comparison condition (comparison of after the AAI program to after the control condition);

† p < 0.10;

* p < 0.05;

** p < 0.01;

*** *p < 0.001*.

a *Unpublished thesis*.

b *Significant at one of three time points. The largest p-value was used if multiple tests evaluated the same outcome*.

**Table 4:** **Assessment measures**.

**First author**	**Year**	**Type**	**Standardized instrument(s)**	**Raters/informants**		
				**Research staff**	**Parent**	**Self**
Woolley^a^	2004	Survey	BDI-II, STAI, YOQ, YSR	x	–	x
McCullough^a^	2011	Survey	CRIES-13, HABS	–	–	x
Hamama	2011	Survey	PCL-C, SCESD	–	–	x
Dietz	2012	Survey	TSCC	–	–	x
Kemp	2013	Survey	BAI, BDI, CBCL, CDI, TSCC	–	–	x
Murrow^a^	2013	Survey, observation	DESSA	x	x	–
Nevins	2013	Survey	BDI-II, MSSS, PCL-C, QOLI, RSES	–	–	x
Lass-Hennemann	2014	Survey, physiological	PANAS, STAI	x	–	x
Newton^a^	2014	Interview	–	–	–	x
Balluerka	2014	Survey	CaMiR	–	–	x

*Standardized instruments and raters refer to survey, interview, and observational data only (not physiological). X, measure used; −, measure not used; BAI, Beck Anxiety Inventory; BDI, Beck Depression Inventory; CaMiR, Cartes: Modèles Individuels de Relation; CBCL, Child Behavior Checklist; CDI, Children's Depression Inventory; CRIES-13, Revised Child Impact of Event Scale; DESSA, Devereux Student Strengths Assessment; HABS, Human-Animal Bond Scale; MSSS, Modified Social Support Survey; PANAS, Positive and Negative Affect Schedule; PCL-C, PTSD Checklist – Civilian Version; QOLI, Quality Of Life Inventory; RSES, Response to Stressful Experiences Scale; SCESD, Short Center for Epidemiologic Studies Depression Scale; STAI, State-Trait Anxiety Inventory; TSCC, Trauma Symptom Checklist for Children; YOQ, Youth Outcome Questionnaire; YSR, Youth Self-Report*.

#### Sample size and characteristics

Sample sizes ranged from 1 to 153 participants, with half of the studies (*n* = 5) having a relatively small sample size of ≤11 participants. For studies with more than one participant, the percentage of males ranged from 0 to 83%, with males making up 20.2% (74 of 366 participants) of the total sample across the 10 studies.

Three studies were conducted with adults (*n* = 87), and seven studies concentrated on children and adolescents (*n* = 279). Of the three adult studies, only one reported participant age and it was a case study with only one participant and horses (Nevins et al., 2013). The other two studies specified instead that the participants were students with dogs (*N* = 80; Lass-Hennemann et al., 2014) or veterans with service dogs (*N* = 6; Newton, 2014). All studies on children and adolescents reported the age range of their participants; however only five reported the mean age or enough information to calculate it, and only two reported the standard deviation or enough information to calculate it. Using the information provided, the mean age of child and adolescent participants was 12.3 years (range: 4–18, *SD* = 1.93).

#### Diagnosis

Participants were exposed to a range of traumas. Two of three studies with adults included war veterans with a prior community diagnosis of PTSD (Nevins et al., 2013; Newton, 2014). One was a case study with one veteran and horses (Nevins et al., 2013) and the other was a small study with six participants with service dogs (Newton, 2014). The third adult study was a laboratory-based, experimental study with dogs, in which healthy participants were exposed to traumatic video content (Lass-Hennemann et al., 2014).

The seven studies on children and adolescents focused on family violence. None of these studies included participants with a prior diagnosis of PTSD. Most included a combination of physical abuse, sexual abuse, and/or unspecified trauma. Only one study focused on a specific type of abuse, including dogs in school for 30 children who had experienced sexual abuse (Kemp et al., 2013).

A subset of participants in one dog study experienced no trauma (Hamama et al., 2011). It compared a treatment group of nine children with teacher-reported trauma who participated in AAI to a control group of nine children *without* teacher-reported trauma who did *not* participate in AAI. This unequal comparison was further complicated by the fact that a subset of participants in each condition did not qualify for PTSD on the PTSD Checklist for Children (PCL-C; 2 of 9 in treatment group; 6 of 9 in control group). Given the high proportion of children without PTSD in the control group, and the fact that the study did not report separate results for the three individuals who had experienced trauma, the results from the control group in this study were not included in the final review.

#### Study design

Half of the studies included a comparison condition (*n* = 5), while the others looked only at the treatment condition, using a pre-post design (*n* = 4) or retrospective interviews (*n* = 1). The comparison conditions included one study with a within-participant, waitlist to treatment (AB) design and four studies with between-participants comparisons against a waitlist with treatment as usual or the AAI procedure without an animal. Only one of the between-participants studies used random assignment to condition, conducting data collection a laboratory setting with dogs (Lass-Hennemann et al., 2014). Only one study included a follow-up assessment; it was a case study of one veteran and horses with measures at 2, 4, 6, and 12 weeks post-AAI (Nevins et al., 2013).

#### Assessment type

Surveys were the most frequent means of assessment (*n* = 9; Table 4). Responses were predominantly self-report, but also included reports from parents and a treatment facility staff member. In addition to surveys, one study included behavioral observations of nine participants during therapy sessions, counting how many times they approached the therapy dog (Murrow, 2013). Another study assessed the physiological arousal of 80 female participants, measuring their blood pressure, heart rate and cortisol level when with a dog, stuffed dog, person, or nothing (Lass-Hennemann et al., 2014). One study was based on qualitative interviews alone, evaluating six veterans with service dogs (Newton, 2014). No studies incorporated blinded observational measures of participant outcomes.

### Outcomes of AAI for PTSD

To achieve the third aim of this review, we synthesized the study outcomes. Although the designs and assessments of the 10 studies were varied, key outcomes were identified and categorized according to the number of studies in which they were reported. Table 5 reports effect sizes and mean percent change from before to after AAI for the most commonly reported outcomes in quantitative studies.

**Table 5:** **Percent change and effect size of most commonly reported outcomes in quantitative studies**.

**Outcome measures**	**First author**	**Year**	**Pre-AAI vs. Post-AAI**			**Post-AAI vs. post-control**		
			***p***	**Mean % change**	***d***	**Control**	***p***	***d***
**DEPRESSION**								
BDI	Kemp	2013	< 0.001	−53	1.31	Waitlist	< 0.01	—
	Nevins	2013	—	−44	—			
	Woolley^a^	2004	**ns**	1^b^, −47^c^	0.01^b^, 0.44^c^	Waitlist	—	0.01^b^, 0.38^c^
CDI	Kemp	2013	< 0.001	−72	1.97	Waitlist	< 0.01	—
SCESD	Hamama	2011	0.06	−19	0.47			
TSCC	Dietz	2012	< 0.001	−46	0.92	No animal	< 0.001	0.53
	Kemp	2013	< 0.001	−69	2.91	Waitlist	< 0.01	—
**PTSD SYMPTOMS**								
CRIES-13	McCullough^a^	2011	< 0.05	−13	0.34			
PCL-C	Hamama	2011	< 0.05	−22	0.70			
	Nevins	2013	—	−34	—			
TSSC	Dietz	2012	< 0.001	−39	0.86	No animal	**ns**	0.53
	Kemp	2013	< 0.001	−80	3.77	Waitlist	< 0.01	—
**ANXIETY**								
BAI	Kemp	2013	< 0.001	−65	2.09	Waitlist	< 0.01	—
STAI-S	Woolley	2004	**ns**	−21	0.61	Waitlist	—	0.51
TSSC	Dietz	2012	< 0.001	−43	0.80	No animal	< 0.001	0.64
	Kemp	2013	< 0.001	−58	2.63	Waitlist	< 0.01	—

–*, not reported or not enough information to calculate; d, Cohen's d effect size; ns, not significant*.

a *Thesis*.

b *Post-AAI is the full BDI before the last session*.

c *Post-AAI is the brief BDI after the last session*.

#### Depression

The most commonly reported outcome from AAI for trauma was a reduction in depression symptoms in six out of 10 studies. Four different instruments were used to assess depression, including Beck's Depression Inventory (BDI), the Children's Depression Inventory (CDI), the Short Center for Epidemiologic Studies Depression Scale (SCESD), and the Trauma Symptom Checklist for Children (TSCC). One study also included qualitative self-report. Outcomes included reduced depression following AAI, compared to before the AAI (*n* = 5) and after the comparison condition (*n* = 2). One study found reduced depression from before to after individual AAI sessions with farm animals for 21 participants (−51% mean change, Woolley, 2004). However, changes over the course of the program compared to baseline were only evidenced *after* the last session (−47% mean change) but not *before* the last session (+1% mean change). Thus, in this study the changes appear to be short-term, whereas a case-study on a war veteran with horses found lasting changes at 12 weeks post-AAI (−44% mean change, Nevins et al., 2013). Taken together, there was variability in the timing magnitude of changes, with the mean percent change from before to after AAI ranging from −19 to −72%. Effect sizes ranged from small to large.

#### PTSD symptoms

The second most commonly reported outcome from AAI for trauma was a reduction in PTSD symptoms in five out of 10 studies. Three different instruments were used to assess PTSD symptoms, including the Children's Revised Impact of Event Scale (CRIES), the PTSD Checklist—Civilian Version (PCL-C), and the Trauma Symptom Checklist for Children (TSCC). Outcomes included reduced PTSD symptoms following AAI, compared to before the AAI in five studies. These changes were significant compared to the comparison condition in one study of 30 participants with horses (Kemp et al., 2013) but not in another study of 153 participants with dogs (Dietz et al., 2012). These two studies also examined dissociation as a separate symptom using the TSCC and both found significant decreases in dissociation symptoms following AAI, compared to before the AAI and after the comparison condition. There was a high variability in the magnitude of changes, with the mean percent change from before to after AAI ranging from −13 to −80% across the five studies. Effect sizes ranged from small to large.

#### Anxiety

Another common finding was reduced anxiety in four studies. Three different survey instruments were used to assess anxiety, including the State-Trait Anxiety Inventory (STAI), the Trauma Symptom Checklist for Children (TSCC), and Beck's Anxiety Inventory (BAI). Outcomes in two studies included reduced anxiety following AAI, compared to before the AAI and to the comparison condition (Dietz et al., 2012; Kemp et al., 2013). In a study on AAI with farm animals, results showed a short-term effect on anxiety from before to after individual AAI sessions (−27% mean change, Woolley, 2004). However, changes over the course of the program compared to baseline were only evidenced *after* the last session (−21% mean change) but not *before* the last session (+12% mean change). The final study showed that 80 healthy, female participants reported feeling less anxious watching a traumatic video with a dog or a person, compared to a stuffed dog or alone. However, physiological measures (i.e., cortisol, heart rate and blood pressure) showed no differences based on condition (Lass-Hennemann et al., 2014). The mean percent change in anxiety from before to after AAI ranged from −21% to −65% across the four studies. Effect sizes ranged from small to large.

#### Social outcomes

A variety of outcomes were relevant to participants' social environment and social competencies. Among these, one study with farm animals reported a 20% increase (*d* = 0.50) in attachment security in 21 adolescents from before to after AAI as measured by the Cartes: Modèles individuels de Relation (CaMiR) questionnaire (Balluerka et al., 2014), and one with dogs reported non-significant improvements in social-emotional competencies from before to after AAI in nine children (+8%, *d* = 0.40) as measured by the Devereux Student Strength Assessment (Murrow, 2013). Qualitative reports from six veteran service dog recipients indicated an increase in involvement with helping others and feelings of social support after receiving their service dogs. Conversely, they also acknowledged that owning a service dog can bring social difficulties, such as being denied access to public places or lack of respect for the dog as a working animal (Newton, 2014).

#### Sleep

Two studies assessed outcomes related to sleep. One war veteran participating in a case-study reported a lasting increase in nightly sleep duration that continued for 3 months after the AAI program (Nevins et al., 2013). Another study with six war veterans reported drops in the frequency of nightmares when living with service dogs (Newton, 2014).

#### Child functioning

Two studies specifically addressed areas of child functioning. One study reported a 63% reduction in problem behaviors among 30 children and adolescents following AAI with horses, compared to before AAI (*d* = 2.26) as measured by the Child Behavior Checklist (Kemp et al., 2013). Another study of therapy sessions with a dog reported increases in global scores of behavioral functioning from before to after AAI as measured by staff on the Youth Outcome Questionnaire to measure treatment progress (+15% mean change, *d* = 0.41), but not in the aggregated scores of the 11 participants on the Youth Self-Report to measure problem behaviors (Woolley, 2004).

#### Quality of life

A final group of findings addressed constructs related to quality of life. A war veteran case study reported increases in satisfaction with quality of life (+180% change) and decreases in dissatisfaction with quality of life (−93% change) from before to after AAI with horses (Nevins et al., 2013). Outcomes related to self-efficacy included significant improvements in coping with stressful life events during AAI with dogs (+7% mean change, *d* = 0.14; Hamama et al., 2011) and increased resilience-focused behaviors and processes in the case-study with a war veteran and horses (35% increase; Nevins et al., 2013). Qualitative interview data indicated reduced fear of public spaces and less use of psychotropic medications in six war veterans with service dogs (Newton, 2014). In the laboratory-based study, dogs helped reduce a subjective drop in negative affect after the participants had watched the traumatic video, but there was no increase in positive affect (Lass-Hennemann et al., 2014). Other studies with dogs reported reductions in anger (−41% mean change, *d* = 0.68; Dietz et al., 2012) and increases in well-being (+10% mean change, *d* = 0.24; Hamama et al., 2011) from before to after AAI.

## Discussion

We conducted a systematic review to synthesize the empirical literature on AAI for individuals who have experienced trauma, including PTSD. The exhaustive search procedure resulted in 10 studies, including six peer-reviewed journal articles and four theses. There has been a recent growth in the number of studies on AAI for trauma, with all but one study published in the last 5 years. Results support short-term, subjective benefits of AAI for trauma, including reduced depression, PTSD symptoms, and anxiety. Effect sizes ranged from small to large. Intervention procedures and research designs varied greatly, evidencing the preliminary nature of research in this area. The field of research is international and interdisciplinary, with a global range of corresponding author countries and diverse journal disciplines. The broad range of outlets highlights the need for systematically collecting and synthesizing the literature in one place, which was the purpose of this review. Each study was reviewed to achieve three key aims: (a) describe the characteristics of AAI for trauma, (b) evaluate the state of the evidence base to provide recommendations for further research, and (c) summarize the reported outcomes of AAI for trauma.

### Characteristics of AAI for trauma

To achieve the first aim of the review, several elements of AAI were examined in each study, including terminology, animals, setting, interventionist, format, activities, and duration. Across the 10 studies reviewed, five different terms were used to identify AAI, with “animal-assisted therapy” being the most common term used in four studies. The field of human-animal interaction has experienced a push to unify terminology. The core terminology are undisputed; however, the organizational structure of the terms has been met with some dispute regarding whether Animal-Assisted Activities is a sub-category of Animal-Assisted Intervention or its own separate entity. Based on our review of the literature, we recommend the continued use of the term *Animal-Assisted Therapy* to signify individualized, goal-directed treatment, *Animal-Assisted Education* for individualized, goal-directed education, *Animal-Assisted Activities* for unstructured, enrichment activities, and *Service Animals* for trained animals living in the home and with individuals throughout their daily life (Kruger and Serpell, 2010; IAHAIO, 2013). The inconsistency in terminology across the reviewed studies may be a function of the diverse and nascent nature of the field. Given the diversity of reviewed programs, we recommend that future studies follow a consistent taxonomy with the spectrum term AAI and its sub-categories. Consistent terminology will enable more cohesive and productive research as well as better community understanding of the definition and function of AAI, which will influence both practice and policy.

Despite inconsistent terminology, all studies were consistent in that they each presented an animal for individuals who had experienced trauma. The most common animal species were dogs and horses, with a small subset of studies also including a range of farm animals. The settings with dogs were more variable than those with horses or farm animals, which were limited to outdoor farms and riding centers. Interventionists included personnel with a range of backgrounds in both human-focused (e.g., psychologist, social worker) and animal-focused (e.g., veterinarian, animal-handler) domains. Limited information regarding specific AAI training for interventionists was provided. We recommend that future studies provide specific details about both animal and interventionist background, training, and experience with AAI.

The level of detail regarding AAI procedures was often insufficient to enable replication. From the information provided, protocols varied widely, even across studies with the same species. Formats included both individual- and group-based intervention. The duration of weekly AAI programs ranged from 1 to 12 weeks, with contact time ranging from 20 min to 36 h over the course of the AAI program. No studies used fidelity checklists to assess treatment integrity. Only one study experimentally evaluated a procedural component of the intervention. Findings revealed that positive outcomes were greater when dogs were meaningfully incorporated into child and adolescent group therapy through stories, compared to when they were simply present in the room without story integration (Dietz et al., 2012). No study used a published treatment manual, which is a critical component of establishing evidence-based interventions (Foa et al., 1997). Replicable protocols are recommended to enable evaluation of generalizability in research and consistent implementation in community practice (Cukor et al., 2009). Given the procedural variability across studies, it appears that AAI for trauma is at present not well defined and in need of technique refinement and protocol standardization.

### Assessing AAI for trauma

To achieve the second aim of the review—to evaluate the state of the evidence base—we reviewed the methodology of the included studies. There was a wide variability across the studies with respect to sample size and characteristics, study design, and assessments. Sample sizes ranged from a case study design with one participant to a larger study with 153 participants. Participants included children, adolescents, and adults. Not all studies reported descriptive statistics regarding participant age, which should be more carefully reported in further research.

The type of trauma varied across studies, with the most common type being child abuse. Despite a growing number of AAI programs targeting military veterans, only two studies included this population; one was a case study and the other was an unpublished thesis. It appears that there is a gap in the empirical literature on the effects of animals for veterans. Given the broad range of trauma types, it would be valuable for further research to investigate and develop treatments that are best adapted to specific experiences. For example, factors that may influence AAI format and outcomes could include the timing of the trauma (e.g., recent vs. distant past), the age group of participants during treatment and during trauma (e.g., child vs. adult), and the cause of the trauma (e.g., war, physical assault, sexual abuse, or witnessing violence). Given predominantly small sample sizes in existing studies, it is not possible to explore individual differences to determine the characteristics of people who benefit from AAI. In the future, larger studies can be used to distinguish the profiles of individuals who are most likely to benefit from AAI. This will enable efficient and effective allocation of AAI services.

The trajectory of research to establish specialized AAI programs for trauma is in its very early stages. The current body of research consists predominantly of small, pre-post studies. These types of studies are recommended as the first step in PTSD treatment research, as a means of documenting feasibility, safety, and potential treatment benefits (Rosenberg et al., 2011). Although the existing studies' successes imply feasibility and safety, they do not directly address or report on these outcomes. Further studies should specifically assess and report outcomes for feasibility and safety, in addition to treatment benefits. This stage is generally followed by the development of standardized, manual procedures with fidelity checklists and subsequent randomized clinical trials. However, we recognize that the steps to develop evidence-based, complementary, and integrative treatments are often not linear and there may be concurrent pursuit of multiple research goals related to the development and evaluation of AAI for trauma (National Center for Complementary and Alternative Medicine, 2011).

One noteworthy area of consideration for further research is the selection of an appropriate control condition. Half of the included studies had no control condition. The other half predominantly used a standard of care control. The standard of care control condition is essential to establish whether there is any effect of AAI, above and beyond current practices or treatment as usual. These designs are important to establish whether or how AAI can add value to existing approaches. Yet while findings from these studies can attribute outcomes to the AAI program as a whole, they do not necessarily evidence any specific role of the animal. If effectiveness trials continue to demonstrate benefits from AAI, follow up studies may begin to compare AAI to a placebo or sham treatment to disentangle the effects of the animal from potential effects due to novelty, expectancy biases, or extraneous treatment components. The challenge in AAI research will be to identify suitable attention controls to isolate these factors.

### Outcomes of AAI for trauma

Although the reviewed studies are diverse and limited, all reported positive outcomes of AAI for individuals who have experienced trauma. It is important to interpret these outcomes as preliminary, given the low level of methodological rigor in many of the studies. The most common finding was reduced depression following AAI. This outcome is consistent with prior research indicating that AAI can reduce depression among individuals in nursing homes and psychiatric hospitals (Souter and Miller, 2007). Reductions in depression may be related to positive perceptions of animals. Indeed the simple presence of an animal has been related to increased instances of smiling and laughing among children (O'Haire et al., 2013) as well as positive social engagement among adults (Hunt et al., 1992; Wood et al., 2005).

The second most commonly reported outcome was reduced PTSD symptom severity. It is unclear whether there are specific symptoms that were targeted in AAI protocols, or whether there are specific symptoms that were most amenable to change from AAI. Reductions in depression may be related to changes in the PTSD symptom of negative alterations in cognition and mood. Another symptom, alterations in arousal and reactivity, may be interrelated with the finding of reduced anxiety following AAI. Previous research has documented an anxiety-reducing effect of animals in many studies (e.g., Shiloh et al., 2003). The reduction in arousal may be due to the comforting soft contact of stroking an animal (Beetz et al., 2012a) or to the centering ability of animals to act as a positive external focus of attention (Gullone, 2000). Exploring the mechanisms of change during AAI offers a complex and open area for ongoing investigation. It will likely require careful manipulation of a set number of interaction variables, such as physical contact, attentional focus, and situational demands.

The duration of positive outcomes was only examined in a case study of one war veteran with horses (Nevins et al., 2013). Follow-up measures in this study indicated lasting benefits; however, another study of AAI sessions for 21 adolescents showed positive benefits only *during* sessions with the farm animals, but not following a week-long period without the animals (Woolley, 2004). Further research should focus specific attention on the nature and timing of positive outcomes, as well as the duration required to achieve them.

It would also be informative to document differential outcomes based on the type of interaction and activity with each animal. For example, the largest study reviewed (*N* = 153 children and adolescents) demonstrated that AAI with dogs resulted in greater positive outcomes if the dog was incorporated into the therapy setting by telling stories from the dog's perspective, rather than simply being present during sessions (Dietz et al., 2012). In the development of effective AAI manuals, it may be fortuitous to test variable treatment components such as AAI activities or animal species. Example variables might include the format of treatment (e.g., group vs. individual), type of contact (e.g., stroking a dog vs. watching a dog), or type of activity (e.g., mounted horse riding vs. un-mounted activities such as grooming). These evaluative processes are critical in the early stages of intervention development and piloting, particularly for complex interventions with multiple components (Craig et al., 2008).

Finally, it is notable that no outcomes were reported related to animal welfare. We hypothesize that a high level of animal welfare is necessary to achieve positive outcomes from AAI. It is important to document the standard of care required and provided to enable consistent replication as well as to highlight animal care as an important component of a successful and ethical AAI program.

### Risk of bias and future directions

The assessments in the reviewed research were predominantly self-report. This is a critical format to capture individual perceptions related to depression, anxiety, and quality of life following trauma. Further studies should corroborate self-reported findings with measures that have a lower risk of bias, such as blinded behavioral observation or physiological assessment. For example, in addition to asking a person about nightmares and sleep quality, it would also be informative to track sleep patterns and arousal via telemetric monitoring devices that can be worn comfortably at night. Advances in technology will enable high-quality, comprehensive assessments from multiple angles to address core PTSD symptoms in addition to comorbid disorders and overall functioning.

Critics of new intervention research may suggest that positive findings are due to a publication bias or file drawer problem, whereby negative or non-significant positive findings are filed away rather than published. To address this possibility, we included both published and unpublished work in our review. Positive outcomes were reported in both categories. However, the effect sizes in published studies were larger than those in unpublished studies. Given the preponderance of methodological weaknesses in the unpublished theses, it is unclear whether the lack of publication is due to study design or findings. What is clear is that the field of research is in a nascent stage, and further inquiry over time will be necessary to truly elucidate a potential publication bias.

Another form of potential bias is researcher expectancy bias. This may be particularly salient for studies in which the researchers designed and conducted the study in addition to providing the intervention. For example, study authors in some cases included an animal handler or other AAI personnel. Independence between the research team and the service providers may lend more credibility to study findings. Once a larger number of quantitative studies have been conducted, it will also be possible to use more sophisticated techniques to evaluate internal and external risk of biases, such as *p*-curves (Simonsohn et al., 2014) or funnel plots (Egger et al., 1997).

## Conclusion

There has been a recent growth in the number of studies examining AAI for trauma. Results have been predominantly positive, showing short-term improvements in depression, PTSD symptoms, and anxiety. A review of the methodology indicates that research in this area is in its very early stages. Given the preliminary nature of the data, we conclude that at present AAI shows promise as a complementary technique, but should not be enlisted as the first line of primary treatment for trauma. Further research is needed to better understand the nature of outcomes for different types of trauma, to directly evaluate feasibility and compliance, to manualize evidence-based AAI treatment protocols, and to evaluate generalizable outcomes in larger community samples.

## Author contributions

MO conceptualized and designed the project, obtained funding for the review, conducted the literature research, analyzed and interpreted data, and participated in the writing of the manuscript. NG analyzed and interpreted data, provided summaries of reviewed articles, conducted statistical analysis, and participated in the writing of the manuscript. AK conducted literature research, analyzed data, and participated in the writing of the manuscript. All authors contributed to and have approved the final manuscript.

### Conflict of interest statement

The authors declare that the research was conducted in the absence of any commercial or financial relationships that could be construed as a potential conflict of interest.

## References

[B1] American Psychiatric Association (2000). Diagnostic and Statistical Manual of Mental Disorders: DSM-IV-TR. Washington, DC: American Psychiatric Association.

[B2] Balluerka N. Muela A. Amiano N. Caldentey M. A. (2014). Influence of animal-assisted therapy (AAT) on the attachment representations of youth in residential care. Child. Youth Serv. Rev. 42, 103–109. 10.1016/j.childyouth.2014.04.007

[B3] Banks M. R. Banks W. A. (2002). The effects of animal-assisted therapy on loneliness in an elderly population in long-term care facilities. J. Gerontol. A Biol. Sci. Med. Sci. 57, M428–M432. 10.1093/gerona/57.7.M428 12084804

[B4] Barker S. B. Pandurangi A. K. Best A. M. (2003). Effects of animal-assisted therapy on patients' anxiety, fear, and depression before ECT. J. ECT 19, 38–44. 10.1097/00124509-200303000-00008 12621276

[B5] Becker C. B. Zayfert C. Anderson E. (2004). A survey of psychologists' attitudes towards and utilization of exposure therapy for PTSD. Behav. Res. Ther. 42, 277–292. 10.1016/S0005-7967(03)00138-4 14975770

[B6] Beetz A. Julius H. Turner D. Kotrschal K. (2012a). Effects of social support by a dog on stress modulation in male children with insecure attachment. Front. Psychol. 3:352. 10.3389/fpsyg.2012.00352 23162482 PMC3498889

[B7] Beetz A. Uvnäs-Moberg K. Julius H. Kotrschal K. (2012b). Psychosocial and psychophysiological effects of human-animal interactions: the possible role of oxytocin. Front. Psychol. 3:234. 10.3389/fpsyg.2012.00234 22866043 PMC3408111

[B8] Bernabei V. De Ronchi D. La Ferla T. Moretti F. Tonelli L. Ferrari B.. (2013). Animal-assisted interventions for elderly patients affected by dementia or psychiatric disorders: a review. J. Psychiatr. Res. 47, 762–773. 10.1016/j.jpsychires.2012.12.014 23369337

[B9] Bomyea J. Lang A. J. (2012). Emerging interventions for PTSD: future directions for clinical care and research. Neuropharmacology 62, 607–616. 10.1016/j.neuropharm.2011.05.028 21664365 PMC3626560

[B10] Craig P. Dieppe P. Macintyre S. Michie S. Nazareth I. Petticrew M. (2008). Developing and evaluating complex interventions: the new Medical Research Council guidance. BMJ 337:a1655. 10.1136/bmj.a1655 18824488 PMC2769032

[B11] Cukor J. Spitalnick J. Difede J. Rizzo A. Rothbaum B. O. (2009). Emerging treatments for PTSD. Clin. Psychol. Rev. 29, 715–726. 10.1016/j.cpr.2009.09.001 19800725

[B12] Dietz T. J. Davis D. Pennings J. (2012). Evaluating animal-assisted therapy in group treatment for child sexual abuse. J. Child Sex. Abus. 21, 665–683. 10.1080/10538712.2012.726700 23194140

[B13] Egger M. Davey Smith G. Schneider M. Minder C. (1997). Bias in meta-analysis detected by a simple, graphical test. BMJ 315, 629–634. 10.1136/bmj.315.7109.629 9310563 PMC2127453

[B14] Filan S. L. Llewellyn-Jones R. H. (2006). Animal-assisted therapy for dementia: a review of the literature. Int. Psychogeriatr. 18, 597–611. 10.1017/S1041610206003322 16640796

[B15] Foa E. B. Cashman L. Jaycox L. Perry K. (1997). The validation of a self-report measure of posttraumatic stress disorder: the Posttraumatic Diagnostic Scale. Psychol. Assess. 9:445 10.1037/1040-3590.9.4.445

[B16] Gullone E. (2000). The biophilia hypothesis and life in the 21st century: increasing mental health or increasing pathology? J. Happiness Stud. 1, 293–322. 10.1023/A:1010043827986

[B17] Hamama L. Hamama-Raz Y. Dagan K. Greenfeld H. Rubinstein C. Ben-Ezra M. (2011). A preliminary study of group intervention along with basic canine training among traumatized teenagers: a 3-month longitudinal study. Child. Youth Serv. Rev. 33, 1975–1980. 10.1016/j.childyouth.2011.05.021

[B18] Hidalgo R. B. Davidson J. R. (2000). Posttraumatic stress disorder: epidemiology and health-related considerations. J. Clin. Psychiatry 61(suppl 7), 5–13. 10795604

[B19] Hunt S. J. Hart L. A. Gomulkiewicz R. (1992). Role of small animals in social interactions between strangers. J. Soc. Psychol. 132, 245–256. 10.1080/00224545.1992.9922976

[B20] IAHAIO (2013). The IAHAIO Definitions for Animal-assisted Intervention and Animal-assisted Activity and Guidelines for Wellness of Animals Involved. Available online at: http://www.iahaio.org/new/fileuploads/8000IAHAIO%20WHITE%20PAPER%20TASK%20FORCE%20-%20FINAL%20REPORT%20-%20070714.pdf

[B21] Kemp K. Signal T. Botros H. Taylor N. Prentice K. (2013). Equine facilitated therapy with children and adolescents who have been sexually abused: a program evaluation study. J. Child Fam. Stud. 23, 558–566. 10.1007/s10826-013-9718-1

[B22] Kessler R. C. Sonnega A. Bromet E. Hughes M. Nelson C. B. (1995). Posttraumatic stress disorder in the National Comorbidity Survey. Arch. Gen. Psychiatry 52, 1048–1060. 10.1001/archpsyc.1995.03950240066012 7492257

[B23] Kruger K. A. Serpell J. A. (2010). Animal-assisted interventions in mental health: definitions and theoretical foundations, in Handbook on Animal-assisted Therapy: Theoretical Foundations and Guidelines for Practice, ed Fine A. H. (San Diego, CA: Academic Press), 33–48.

[B24] Lakens D. (2013). Calculating and reporting effect sizes to facilitate cumulative science: a practical primer for t-tests and ANOVAs. Front. Psychol. 4:863. 10.3389/fpsyg.2013.00863 24324449 PMC3840331

[B25] Lass-Hennemann J. Peyk P. Streb M. Holz E. Michael T. (2014). Presence of a dog reduces subjective but not physiological stress responses to an analog trauma. Front. Psychol. 5:1010. 10.3389/fpsyg.2014.01010 25250009 PMC4158977

[B26] Liberati A. Altman D. G. Tetzlaff J. Mulrow C. Gøtzsche P. C. Ioannidis J. P. A.. (2009). The PRISMA statement for reporting systematic reviews and meta-analyses of studies that evaluate healthcare interventions: explanation and elaboration. BMJ 339:b2700. 10.1136/bmj.b2700 19622552 PMC2714672

[B27] Marr C. A. French L. Thompson D. Drum L. Greening G. Mormon J. (2000). Animal-assisted therapy in psychiatric rehabilitation. Anthrozoos 13, 43–47. 10.2752/089279300786999950

[B28] McCullough L. M. (2011). Effect of Equine-facilitated Psychotherapy on Posttraumatic Stress Symptoms in Youth with History of Maltreatment and Abuse. 72, Prescott Valley, AZ: ProQuest Information & Learning.

[B29] McNicholas J. Collis G. M. (2000). Dogs as catalysts for social interaction: robustness of the effect. Br. J. Psychol. 91, 61–70. 10.1348/000712600161673 10717771

[B30] Moher D. Liberati A. Tetzlaff J. Altman D. G. (2009). Preferred reporting items for systematic reviews and meta-analyses: the PRISMA statement. BMJ 339:b2535. 10.1136/bmj.b2535 19622551 PMC2714657

[B31] Morris S. B. (2007). Estimating effect sizes from pretest-posttest-control group designs. Org. Res. Methods. 11, 364–386. 10.1177/109442810629105919271847

[B32] Murrow B. L. (2013). A Quantitative Exploration into the Effects of the Human and Animal Connection. 3597045 Ph.D., Pacifica Graduate Institute.

[B33] National Center for Complementary Alternative Medicine (2011). Exploring the Science of Complementary and Alternative Medicine: Third Strategic Plan. Washington, DC: Government Printing Office.

[B34] Nevins R. Finch S. Hickling E. J. Barnett S. D. (2013). The Saratoga WarHorse project: a case study of the treatment of psychological distress in a veteran of Operation Iraqi Freedom. Adv. Mind Body Med. 27, 22–25. Available online at: http://www.scopus.com/inward/record.url?eid=2-s2.0-84896058705partnerID=40md5=24c876a42181ccab702fc7c4f80494ed 24067322

[B35] Newton R. (2014). Exploring the Experiences of Living With Psychiatric Service Dogs for Veterans with Posttraumatic Stress Disorder. 1557029 M.A., Adler School of Professional Psychology.

[B36] Nimer J. Lundahl B. (2007). Animal-assisted therapy: a meta-analysis. Anthrozoös 20, 225–238. 10.2752/089279307X224773

[B37] O'Haire M. E. (2013). Animal-assisted intervention for autism spectrum disorder: a systematic literature review. J. Autism Dev. Disord. 43, 1606–1622. 10.1007/s10803-012-1707-5 23124442

[B38] O'Haire M. E. McKenzie S. J. Beck A. M. Slaughter V. (2013). Social behaviors increase in children with autism in the presence of animals compared to toys. PLoS ONE 8:e57010. 10.1371/journal.pone.0057010 23468902 PMC3584132

[B39] Parish-Plass N. (2008). Animal-assisted therapy with children suffering from insecure attachment due to abuse and neglect: a method to lower the risk of intergenerational transmission of abuse? Clin. Child Psychol. Psychiatry 13, 7–31. 10.1177/1359104507086338 18411863

[B40] Rosenberg H. J. Jankowski M. K. Fortuna L. R. Rosenberg S. D. Mueser K. T. (2011). A pilot study of a cognitive restructuring program for treating posttraumatic disorders in adolescents. Psychol. Trauma 3, 94 10.1037/a0019889

[B41] Schottenbauer M. A. Glass C. R. Arnkoff D. B. Tendick V. Gray S. H. (2008). Nonresponse and dropout rates in outcome studies on PTSD: review and methodological considerations. Psychiatry 71, 134–168. 10.1521/psyc.2008.71.2.134 18573035

[B42] Serpell J. A. (2006). Animal-assisted interventions in historical perspective, in Handbook on Animal-assisted Therapy: Theoretical Foundations and Guidelines for Practice, ed Fine A. H. (San Diego, CA, Academic Press), 3–20.

[B43] Shiloh S. Sorek G. Terkel J. (2003). Reduction of state-anxiety by petting animals in a controlled laboratory experiment. Anxiety Stress Coping 16, 387–395. 10.1080/1061580031000091582

[B44] Simonsohn U. Nelson L. D. Simmons J. P. (2014). P-curve: a key to the file-drawer. J. Exp. Psychol. Gen. 143, 534. 10.1037/a0033242 23855496

[B45] Souter M. A. Miller M. D. (2007). Do animal-assisted activities effectively treat depression? A meta-analysis. Anthrozoös 20, 167–180. 10.2752/175303707X207954

[B46] Tedeschi P. Fine A. H. Helgeson J. I. (2010). Assistance animals: their evolving role in psychiatric service applications, in Handbook on Animal-assisted Therapy: Theoretical Foundations and Guidelines for Practice, ed Fine A. H. (San Diego, CA, Academic Press), 421–438.

[B47] Wood L. Giles-Corti B. Bulsara M. (2005). The pet connection: pets as a conduit for social capital? Soc. Sci. Med. 61, 1159–1173. 10.1016/j.socscimed.2005.01.017 15970228

[B48] Woolley C. C. (2004). Changes in Child Symptomatology Associated with Animalssisted Therapy. 3157804 Ph.D., Utah State University.

[B49] Yount R. Ritchie E. C. St Laurent M. Chumley P. Olmert M. D. (2013). The role of service dog training in the treatment of combat-related PTSD. Psychiatr. Ann. 43, 292 10.3928/00485713-20130605-11

